# Association of Adherence to The Mediterranean Diet with Urinary Factors Favoring Renal Lithiasis: Cross-Sectional Study of Overweight Individuals with Metabolic Syndrome

**DOI:** 10.3390/nu11081708

**Published:** 2019-07-24

**Authors:** Rafael M. Prieto, Adrian Rodriguez, Pilar Sanchis, Margalida Morey, Miquel Fiol, Felix Grases, Olga Castañer, Miguel A. Martínez-González, Jordi Salas-Salvadó, Dora Romaguera

**Affiliations:** 1 Laboratory of Renal Lithiasis Research, University Institute of Health Science Research (IUNICS-IdISBa), University of the Balearic Islands, 07122 Palma de Mallorca, Spain; 2 CIBER Fisiopatología de la Obesidad y Nutrición (CIBERObn), Instituto de Salud Carlos III, 28029 Madrid, Spain; 3 Instituto de Investigación Sanitaria de les Illes Balears (IdISBa), University Hospital Son Espases, 07120 Palma de Mallorca, Spain; 4 Cardiovascular Risk and Nutrition (Regicor Study Group), Hospital del Mar Research Institute (IMIM), 08003 Barcelona, Spain; 5 Department of Preventive Medicine and Public Health, University of Navarra, 31009 Pamplona, Spain; 6 Department of Nutrition, Harvard TH Chan School of Public Health, Boston, MA 02115, USA; 7 Human Nutrition Unit, University Hospital of Sant Joan de Reus, 43204 Reus, Spain; 8 Department of Biochemistry and Biotechnology, Faculty of Medicine and Health Sciences, Institut d’Investigació Sanitària Pere Virgili, Rovira i Virgili University, 43003 Tarragona, Spain

**Keywords:** Mediterranean diet, renal lithiasis, urinary crystallization risk

## Abstract

Our purpose was to study the relationship of adherence to the Mediterranean diet (MedDiet) with urinary factors that favor the formation of renal calcium and uric acid stones in overweight and obese participants who had metabolic syndrome. This cross-sectional study examined 267 participants. A well-known MedDiet score (range 0–9) was calculated for each patient, and patients were then categorized has having low (≤3), medium (4–5), or high (≥6) adherence to the MedDiet. Baseline characteristics and urinary parameters were also analyzed. High calcium salt urinary crystallization risk (CaUCR) and high uric acid urinary crystallization risk (UrUCR) were calculated from urinary parameters using pre-defined criteria. More than half of patients with MedDiet scores ≤3 had high UrUCR (55.4%) and high CaUCR (53.8%). In contrast, fewer patients with high adherence (≥6) to the MedDiet had high UrUCR (41.2%) and high CaUCR (29.4%). Relative to those with low adherence, individuals with high adherence had a prevalence ratio (PR) of 0.77 for a high UrUCR (95% CI: 0.46–1.12; *p* for trend: 0.069) and a PR of 0.51 for a high CaUCR (95% CI: 0.26–0.87; *p* for trend: 0.012) after adjusting for age, sex, body mass index, type 2 diabetes, and total energy intake. Our findings indicate that greater adherence to the MedDiet was associated with a reduced CaUCR and a reduced UrUCR. This suggests that adequate dietary management using the MedDiet patterns may prevent or reduce the incidence and recurrence of calcium salt and uric acid renal stones.

## 1. Introduction

Renal lithiasis has a multifactorial etiology. Urine composition is a key factor affecting urine crystal formation, because urine is a metastable liquid containing multiple solutes that can potentially crystallize. A disruption in the balance between urinary promoters and inhibitors of crystallization within the urinary tract, and certain renal macro- and micro-anatomical features can affect crystallization.

The structure and composition of renal calculi vary greatly. They may consist of pure uric acid, pure hydroxyapatite, calcium oxalate with different compositions, and various mixtures of these different compounds. Approximately 70–80% of renal calculi are composed of calcium oxalate or calcium phosphate, about 10–15% are uric acid, 5–10% are struvite, and <1% consist of cysteine or other compounds, such as drug-related crystals [1,2].

The prevalence of renal lithiasis is increasing globally [2,3]. Although the prevalence is 5–10% in Europe, some regions have prevalences up to 15%. The recurrence rates are also variable among different regions, but the overall 10-year recurrence rate is about 50% [1]. The high incidence and recurrence rate of renal lithiasis make this condition a significant economic cost for most societies.

Many studies have suggested that obesity is a significant contributor to renal lithiasis. In particular, there is evidence of a 75% increased incidence of renal lithiasis in overweight and obese patients compared to normal weight counterparts [4]. Metabolic syndrome is also associated with an increased risk of renal lithiasis, as well as systemic diseases such cardiovascular disease and diabetes mellitus. It has also been reported that subjects with metabolic syndrome have an increased incidence of uric acid lithiasis relative to those without metabolic syndrome [5].

Although the etiology of renal lithiasis is multifactorial, diet seems to be an important factor due to its ability to affect urine composition. Some dietary components, beyond liquid intake, can modify important factors that increase the risk of renal lithiasis [6,7,8]. In particular, diet can affect urinary concentrations of calcium and oxalate, alter urinary pH, and modify the urinary levels of other factors that prevent crystal formation and growth (urinary crystallization inhibitors), such as citrate and phytate [7]. Some of these urinary parameters can be considered as risk factors for lithogenic urine, although they differ in the ability to differentially increase the risk of calcium lithiasis and uric acid lithiasis.

The aim of the present cross-sectional study was to assess the association between an overall dietary pattern, specifically the Mediterranean Diet (MedDiet), with urinary parameters known to favor or prevent renal lithiasis in a subset of participants from the PREDIMED-Plus trial who are overweight or obese and have the metabolic syndrome.

## 2. Materials and Methods 

### 2.1. Study Overview and Sample

This study was a cross-sectional analysis of baseline data within the framework of the ongoing PREDIMED-Plus trial, a six-year parallel-group, multicenter, randomized clinical trial of 6874 participants from 23 recruiting centers in Spain. This trial was designed to evaluate the effect of several lifestyle interventions—encouragement of weight loss by adoption of an energy-restricted traditional MedDiet, promotion of physical activity, and provision of behavioral support—on the primary prevention of cardiovascular morbidity and mortality. The PREDIMED-Plus recruitment period was from 5 September 2013 to 31 October 2016. The protocol and a detailed description are reported elsewhere [9] and more information is also available on its website (www.predimedplus.com).

Eligible participants were men and women (aged 55–75 years) who were overweight or obese (BMI ≥ 27 and < 40 kg/m^2^), who met at least three criteria for the metabolic syndrome (abdominal obesity, high blood pressure, high fasting glucose, high triglycerides, and low HDL-cholesterol) [10]. All participants provided written informed consent. The study was approved by the institutional review boards of all recruiting centers and was performed according to the ethical standards of the Declaration of Helsinki. The trial was registered at the International Standard Randomized Controlled Trial (ISRCT: http://www.isrctn.com/ISRCTN89898870) on 24 July 2014. The sub-group of participants in the present study (*n* = 267) were consecutively recruited from 27 May 2014 to 29 June 2016 at University Hospital Son Espases.

### 2.2. Dietary Assessment and Mediterranean Diet Score

Data on dietary intake were collected at baseline. All participants were asked to complete a previously validated 137-item semi-quantitative Spanish-language survey [11,12]. Energy and nutrient intakes were calculated using a computer program based on available information from Spanish food composition tables [13,14].

The Mediterranean diet score (MDS), proposed by Trichopoulou et al. [15], was used to evaluate the consumption of nine foods and nutrients in characteristic of the MedDiet, which were categorized based on the PREDIMED-Plus-wide sex-specific medians. Individuals with consumption of fruits and nuts, vegetables, legumes, cereals, fish, and the ratio of monounsaturated fatty acids to saturated fatty acids (MUFA/SFA) above the median level were each assigned a value of 1 (or 0 if below the median); consumption of meat and dairy products below the median level was each assigned a value of 1 (or 0 if above the median); consumption of ethanol of 10–50 g/day for men and 5–25 g/day for women was assigned a value of 1 (or 0 if outside this range). Thus, the total MDS ranged from 0 to 9, and a higher score indicated greater adherence to the MedDiet. In this study, the participants were categorized as having low (≤3), medium (4–5), or high (≥6) adherence to the MedDiet.

### 2.3. Urine Collection and Analysis

A two-hour spot morning urine sample was collected in the fasting conditions from all participants. Urinary pH (measured using a Crison pH-meter), and the urinary concentrations of calcium (Ca), phosphorus (P), oxalate (Ox), uric acid (Ur), citrate (Cit), and magnesium (Mg) were determined. Ca, P and Mg were measured using inductively coupled plasma tandem atomic emission spectrometry (ICP-AES), Ur was determined using the uricase method, Ox was determined using the oxalate oxidase/peroxidase method (LTA, Milano, Italy), and Cit was determined using an enzymatic assay (Biosystems, Barcelona, Spain). Lithogenic urine was defined by a high calcium salt urinary crystallization risk (CaUCR) or a high uric acid urinary crystallization risk (UrUCR), in accordance with the criteria indicated in Table 1 [16,17,18,19].

### 2.4. Covariates Assessment

Information on sex and age was collected at enrolment. Baseline body weight (kg) and height (cm), measured when the subjects were wearing light clothing and no shoes, was determined in duplicate using a calibrated scale and a wall-mounted stadiometer. Body mass index (BMI) was calculated as weight (kg) divided by square of the height (m). Diabetes was defined as self-reported diagnosis at study inclusion, a baseline HbA1c of 6.5% or more, or use of an antidiabetic medication at baseline, such as insulin, metformin, and other medications.

### 2.5. Statistical Analysis

Continuous variables are expressed as means and standard deviations (SDs), and categorical variables as numbers and percentages. After assessing the normality of data distributions, a one-way ANOVA (continuous variables) or the Chi-squared test (categorical variables) was used to identify statistical differences in baseline characteristics and urinary parameters of the three groups. 

Univariable and multivariable binary logistic regression were used to determine the association of dietary factors with CaUCR and UrUCR. Because of the elevated prevalence of high CaUCR and high UrUCR in our population, odds ratios (ORs) were calculated and then a correction method [20] was applied to determine prevalence ratios (PRs) to reduce exaggerations of the true relative risk (RR). MDS was treated as a categorical variable (low, medium or high) using the low category as the reference. Models were adjusted potential confounders such as age, sex, presence of type 2 diabetes, total daily energy intake, and BMI. The association of each component of the MDS with CaUCR and with UrUCR was also determined using similar models. All statistical analyses were performed using SPSS version 23 software, and a two-sided *p*-value below 0.05 was considered statistically significant.

## 3. Results

### 3.1. Baseline Characteristics and Urinary Parameters

The study population (*n* = 267) had a mean (± SD) age of 65 (± 5) years, a mean BMI of 33 (± 3) kg/m^2^, and consisted of 49.4% women. A total of 27.3% of the population had diabetes, 47.5% had high CaUCR, and 48.3% had high UrUCR. Table 2 shows baseline characteristics and urinary parameters of the groups with low, medium, and high adherence to the MedDiet. Individuals with low adherence were younger and had a higher concentration of urinary uric acid, but the other measured parameters were similar among the three groups.

### 3.2. Calcium and Uric Acid Urinary Crystallization Risk

**Figure 1:**
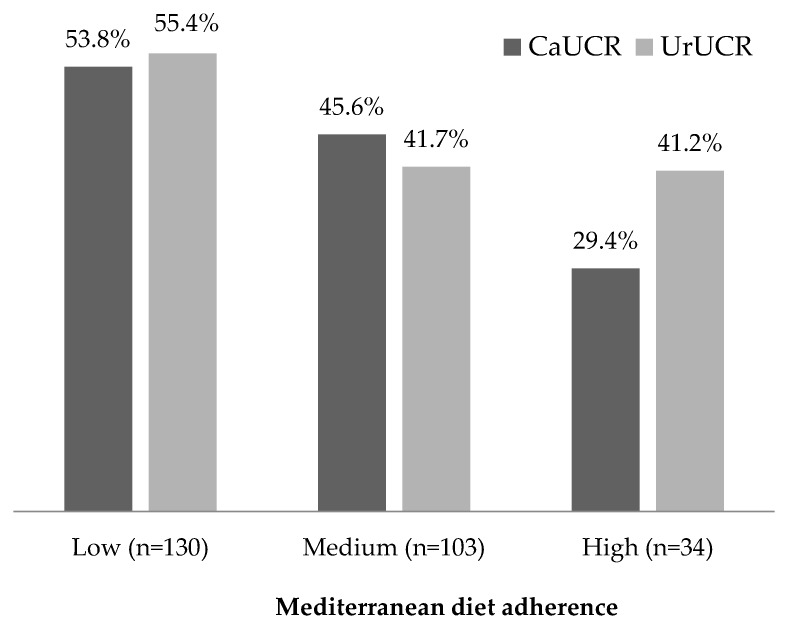
Percentage of subjects with high calcium urinary crystallization risk (CaUCR) and high uric acid urinary crystallization risk (UrUCR) who had low, medium, and high adherence to the MedDiet. Chi-squared tests indicated that high CaUCR had a significant association with diet (*p* = 0.012) and high UrUCR had a marginal association with diet (*p* = 0.079).

Figure 1 shows the percentages of patients with a high CaUCR and a high UrUCR in the three groups. There were clear trends of smaller percentages of patients with each condition as MedDiet adherence increased. Comparison of patients in these different diet adherence categories indicated a significant difference in high CaUCR (53.8%, 45.6%, 29.4%; *p* = 0.012) and a nearly significant difference in high UrUCR (55.4%, 41.7%, 41.2%; *p* = 0.079).

### 3.3. Association between MedDiet Adherence and Urinary Crystallization Risk

Relative to individuals with low MDS scores, those with high scores had a 0.55 PR for a high CaUCR (95% CI = 0.29–0.90; *p* for trend = 0.012) in the unadjusted model and a 0.51 PR for a high CaUCR (95% CI = 0.26–0.87; *p* for trend = 0.012) in the adjusted model (Table 3). Relative to those with low MDS scores, those with high scores had a 0.74 PR for a high UrUCR (95% CI = 0.44–1.08; *p* for trend = 0.042) in the unadjusted model. After adjusting for age, sex, type 2 diabetes, and BMI, this association was no longer significant (*p* for trend = 0.069).

**Figure 2:**
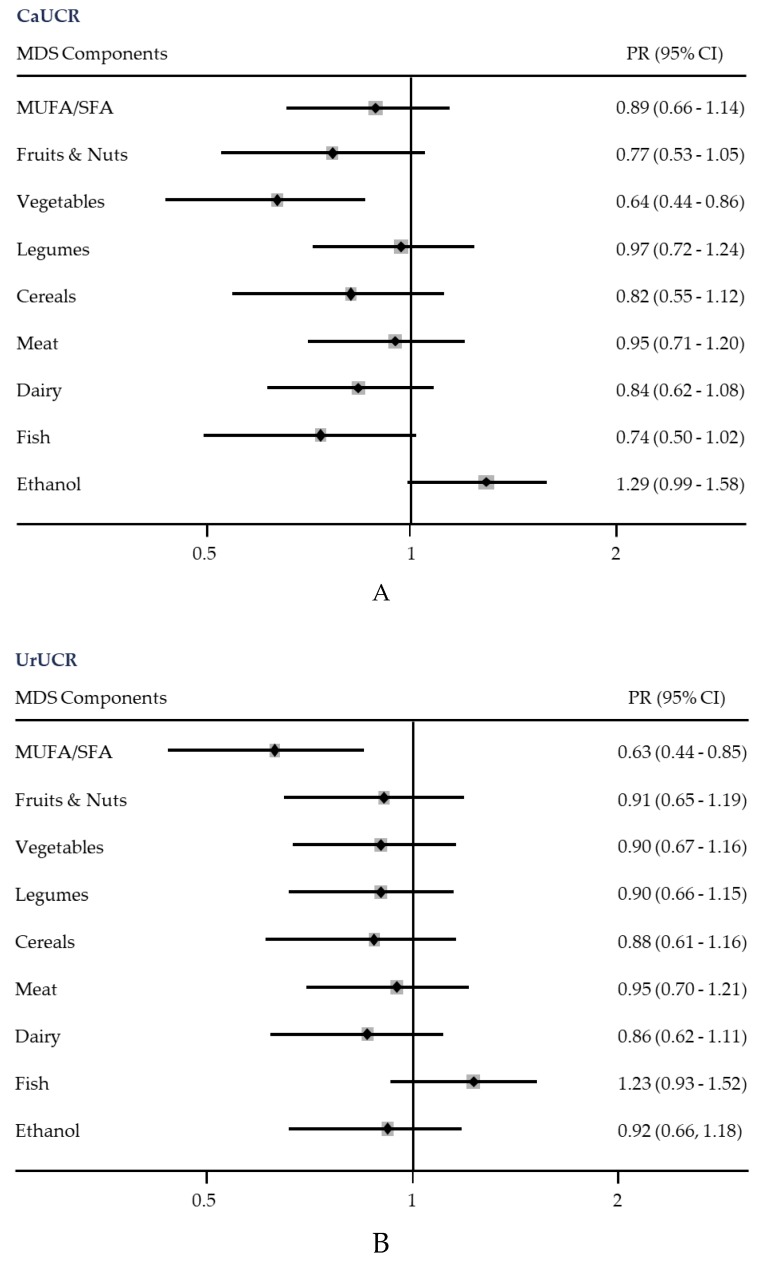
Prevalence ratios (95% CIs) of high calcium urinary crystallization risk (CaUCR) (**A**) and high uric acid urinary crystallization risk (UrUCR) (**B**) with individual components of the Mediterranean diet score (MDS) [15]. Binary logistic regression adjusted for age, sex, type 2 diabetes, body mass index, and energy intake. Each subject received a score of 0 or 1 for each of the nine dietary components (MUFA, mono-unsaturated fatty acid; SFA, saturated fatty acid).

We also analyzed the association between each of the nine components of the MDS with CaUCR (Figure 2A) and UrUCR (Figure 2B). Consuming vegetables was associated with a significantly lower PR of a high CaUCR (0.64, 95% CI = 0.44–0.86), and a high MUFA/SFA ratio was associated with a significantly lower PR of a high UrUCR (0.63, 95% CI = 0.44–0.85). There were trends for associations of moderate ethanol consumption with an increased PR of a high CaUCR, and of fish consumption with a decreased PR of a high CaUCR and an increased PR of a high UrUCR, although these relationships were not statistically significant.

## 4. Discussion

To our knowledge, this is the first study to evaluate the relationship between the MedDiet and lithogenic urine that separately considered CaUCR and UrUCR. The major result of our study of participants who were overweight/obese and had the metabolic syndrome is that high adherence to the MedDiet was associated with lower prevalence of high CaUCR and high UrUCR. For the latter (high UrUCR), when the model was adjusted for age, sex, type 2 diabetes, and BMI, this association was no longer significant (*p* for trend = 0.069) if we consider *p* < 0.05 as a threshold. However, a high or low CaUCR or UrUCR index does not automatically indicate that kidney stones are present in the individual because they are only probabilistic parameters. Many patients with kidney stones may have normal urine composition or healthy people may have metabolic abnormalities in their urine.

A total of 48.3% of our participants had lithogenic urine and high UrUCR. Almost all of those who had high UrUCR (96.1%) had urinary pH levels below 5.5, and the others had uric acid levels above 6.0 mM. A urinary pH below 5.5 is a relevant risk factor for formation of uric acid stones, because it can be easily modified by diet [19]. Subjects who adhered to the MedDiet were marginally less likely to have a high UrUCR (55.4% vs. 41.2%, *p* = 0.079). We also found that the main component of the MDS associated with decreased UrUCR was a high MUFA/SFA ratio. The MUFA/SFA ratio in the MedDiet could indicate low consumption of animal products (which are rich in saturated fats and associated with uric acid stone formation) and/or increased consumption of monounsaturated fats, such as olive oil. This ratio represents a healthy fatty acids intake, i.e. low intake of saturated fat from animal products, such as red and processed meats, and high intake of mono-unsaturated fat from olive oil. High olive oil intake is normally accompanied by high vegetable intake. This ratio, hence, represents not only a healthy intake of fatty acids, but also an overall vegetable-rich, animal-poor diet pattern, which might lead to lower UrUCR. Our results differ from those of the Seguimiento Universidad de Navarra (SUN), a Spanish prospective cohort study of middle-aged university graduates which reported increased risk of any type of renal lithiasis in those who had diets with higher MUFA/SFA ratios, and reduced risk of any type of lithiasis in those with greater consumption of dairy products and vegetables [21]. This may be because we examined older subjects (mean age of ~65 years), who have an approximately two-fold increased risk of uric acid stones [22].

We found that 47.5% of our subjects had high CaUCR using previously established criteria (Table 1). Previous studies demonstrated that nearly 100% of patients who met these criteria were stone-formers [16,17,18]. We also found that subjects who better adhered to the MedDiet had a significantly decreased CaUCR. High consumption of vegetables was the component of the MDS that was most responsible for the decreased CaUCR. This finding is not surprising, because other studies have also reported the protective effect of high consumption of vegetables on any kind of renal lithiasis [23,24]. Vegetables may therefore adjust the urinary pH values and reduce the urinary litogen risk [25].

Although there are not statistically significant differences in the urinary parameters between the three categories (Table 2), we found an overall lower risk for urinary crystallization for those with greater adherence to the MedDiet. These results are consistent with a prospective cohort study of 16,094 participants which found a marked decrease in the incidence of renal lithiasis for those with higher adherence to the MedDiet [21]. However, this study examined the self-reported incidence of any kind of renal lithiasis, and did not measure urinary lithogenic parameters [21].

Other diets, such as DASH-style (Dietary Approach to Stop Hypertension), have been associated with reduced risk of kidney stones [26,27]. The DASH score and the MDS have some similarities: higher intake of fruit, vegetables, legumes and nuts increases the score, while higher intake of meat and meat products decreases the score. One of the differences among these diets is regarding the consumption of dairy products. The DASH diet recommends the consumption of low-fat dairy, whereas the MedDiet is characterized by a moderate intake of overall dairy. 

Our cross-sectional study had some limitations. First, inherent to the cross-sectional design, we cannot exclude reverse causation. Second, we did not analyze 24-h urine samples, and thus could not measure total urinary solute excretion. Third, the urine volume was not considered because we used solute concentration (mg/dL) in CaUCR and UrUCR (Table 1), by which we considered indirectly the hydration of subjects. Fourth, our study population was very specific (age of ~65 years, overweight or obese, and with metabolic syndrome), thus the results cannot be extrapolated to the general population or other population subgroups. However, we had a special interest in analyzing these individuals because a previous systematic review and meta-analysis reported that individuals with metabolic syndrome have a higher prevalence of renal lithiasis [28]. We determined long-term dietary patterns using a validated food frequency questionnaire, but a spot urine sample is more likely to reflect short-term dietary intake. This could potentially lead to lower sensitivity to detect associations with individual foods and nutrients that are consumed less frequently; however, with the use of a dietary score, we might have been able to increase sensitivity to detect such associations. Fifth, because this was an observational cross-sectional study with a modest sample size, we cannot rule out potential residual confounding. The strengths of our study were that we used a previously validated food questionnaire, we had accurate measurements of urinary pH (which is not usually reported in urine analysis), we separately analyzed urinary lithogenic risk based on CaUCR and UrUCR, and we controlled for potential confounders in our statistical models.

In conclusion, our cohort of overweight or obese elderly subjects with metabolic syndrome had a high prevalence of lithogenic urine. Participants with higher adherence to the MedDiet had a lower risk for urinary crystallization by uric acid and calcium salts. Overall, our study together with previous studies provide a rationale for establishment of randomized trials to confirm the protective role of the MedDiet on renal lithiasis. These results suggest that following the MedDiet could reduce the urinary lithogenic risk for uric acid and calcium salts in individuals who are elderly, overweight or obese, and have the metabolic syndrome.

## 5. Conclusions

In conclusion, our cohort of overweight or obese elderly subjects with metabolic syndrome had a high prevalence of lithogenic urine. Participants with higher adherence to the MedDiet had a lower risk for urinary crystallization by uric acid and calcium salts. Overall, our study together with previous studies provide a rationale for establishment of randomized trials to confirm the protective role of the MedDiet on renal lithiasis. These results suggest that following the MedDiet could reduce the urinary lithogenic risk for uric acid and calcium salts in individuals who are elderly, overweight or obese, and have the metabolic syndrome.

## Figures and Tables

**Table 1:** Criteria used to define high calcium salt urinary crystallization risk (CaUCR) and high uric acid urinary crystallization risk (UrUCR), based on [16,17,18,19].

**CaUCR: at least one of four conditions must be present**	
Calcium	>20 mg/dL
Oxalate	>4 mg/dL
Ca/Cit ratio	>0.33
Three or more of these alterations	
pH	<5.5
pH	>6.0
Calcium	>17 mg/dL
Phosphorous	>100 mg/dL
Urate	>60 mg/dL
Citrate	<23 mg/dL
Oxalate	>3 mg/dL
Magnesium	<5 mg/dL
**UrUCR: at least one of two conditions must be present**	
pH	<5.5
Uric acid	>100 mg/dL

**Table 2:** Baseline characteristics and urinary parameters of groups that had low, medium, and high adherence to the MedDiet. Results are expressed as means ± SDs or *n* (%).

	Mediterranean Diet Adherence ^a^			
	Low (*n* = 130)	Medium (*n* = 103)	High (*n* = 34)	*p*-value ^b^
Age (years)	64 ± 5	66 ± 5	65 ± 5	0.023
Sex (% female)	67 (51.5)	48 (46.6)	17 (50)	0.750
DM (%)	37 (28.5)	26 (25.2)	10 (29.4)	0.830
HTA (%)	116 (89.2)	91 (88.3)	29 (85.3)	0.816
BMI (kg/m^2^)	33 ± 3	32 ± 3	32 ± 4	0.093
Energy (kcal/day)	2202 ± 665	2275 ± 609	2287 ±4 36	0.603
pH	5.7 ± 0.7	5.8 ± 0.7	5.8 ± 0.7	0.395
Ur (mg/dL)	55 ± 25	47 ± 23	49 ± 26	0.025
P (mg/dL)	74 ± 40	70 ± 37	59 ± 22	0.131
Cit (mg/L)	639 ± 392	568 ± 347	599 ± 367	0.354
Ox (mg/L)	21 ± 9	21 ± 12	21 ± 9	0.976
Ca (mg/dL)	12 ± 9	11 ± 10	9 ± 5	0.297
Mg (mg/dL)	7 ± 5	7 ± 5	7 ± 3	0.446
Ca/Cit ratio	0.23 ± 0.23 0.20 (0.11–0.29) *	0.26 ± 0.26 0.24 (0.12–0.45) *	0.17 ± 0.12 0.19 (0.07–0.22) *	0.148

DM, diabetes mellitus; HTA, hypertension; BMI, body mass index; Ur, Uric acid; P, phosphorous; Cit, citrate; Ox, oxalate; Ca, calcium; Mg, magnesium; Ca/Cit ratio, calcium to citrate ratio; ^a^ Mediterranean diet adherence was based on the Mediterranean Diet Score (MDS) [15]. Low: MDS ≤ 3; Medium: MDS = 4 or 5; High: MDS ≥ 6. ^b^ One-way ANOVA for continuous variables; Chi-squared test for categorical variables. * Median (interquartile range).

**Table 3:** Prevalence ratios (95% CIs) for high CaUCR and high UrUCR in groups that had low, medium, and high adherence to the MedDiet.

		Mediterranean Diet Adherence ^a^			
		Low (*n* = 130)	Medium (*n* = 103)	High (*n* = 34)	*p* for trend
CaUCR	Model 1 ^b^	ref.	0.85 (0.62, 1.09)	0.55 (0.29, 0.90)	0.012
	Model 2 ^c^	ref.	0.87 (0.63, 1.12)	0.51 (0.26, 0.87)	0.012
UrUCR	Model 1 ^b^	ref.	0.75 (0.54, 0.99)	0.74 (0.44, 1.08)	0.042
	Model 2 ^c^	ref.	0.76 (0.54, 1.00)	0.77 (0.46, 1.12)	0.069

CaUCR, calcium urinary crystallization risk; UrUCR, uric acid urinary crystallization risk. ^a^ Low: MDS ≤ 3; Medium: MDS = 4 or 5; High: MDS ≥ 6. ^b^ Model 1: Crude binary logistic regression. ^c^ Model 2: Binary logistic regression with adjustment for age, sex, type 2 diabetes, body mass index and energy intake.
